# Vascular Responses following Light Therapy: A Pilot Study with Healthy Volunteers

**DOI:** 10.3390/jcm12062229

**Published:** 2023-03-13

**Authors:** Adam Saloň, Bianca Steuber, Ruslan Neshev, Karin Schmid-Zalaudek, Patrick De Boever, Eva Bergmann, Rainer Picha, Per Morten Fredriksen, Benedicta Ngwechi Nkeh-Chungag, Nandu Goswami

**Affiliations:** 1 Gravitational Physiology and Medicine Research Unit, Division of Physiology, Otto Loewi Research Center, Medical University of Graz, 8036 Graz, Austria; 2 Faculty of Health and Social Sciences, Inland Norway University of Applied Science, 2624 Lillehammer, Norway; 3 Centre for Environmental Sciences, Hasselt University, 3500 Hasselt, Belgium; 4 Meditation Center in Silkeborg, Moerksoevej 71, 8600 Silkeborg, Denmark; 5 Rehabilitation Center for Cardiovascular Disease, 8061 St. Radegund, Austria; 6 Department of Biological and Environmental Sciences, Faculty of Health Sciences, Walter Sisulu University PBX1, Mthatha 5117, South Africa; 7 College of Medicine, Mohammed Bin Rashid University of Medicine and Health Sciences, Dubai P.O. Box 505055, United Arab Emirates

**Keywords:** cardiovascular health, healthy volunteers, hemodynamics, retinal imaging, phototherapy, light therapy

## Abstract

(1) Background: Studies have reported the effectiveness of light therapy in various medical conditions. Our pilot study aimed to assess the effect of Maharishi light therapy (MLT) on physiological parameters, such as the heart rate (HR), HR variability (HRV), blood pressure (BP), BP variability (BPV), and the retinal microvasculature of healthy participants; (2) Methodology: Thirty (14 males and 16 females) healthy, non-smoking participants between 23 and 71 years old (46 ± 18 years) were included in this randomized crossover study. Each participant was tested with a placebo (using LED light) and gem lights, 24 h apart. Hemodynamic parameters were recorded during the session, and 24 h heart rate and BP levels were assessed via mobile devices. Retinal vascular responses were captured with fundus images and the subsequent analysis of retinal vessel widths. A linear model, using repeated measures ANOVA, was used to compare the responses across the sexes and to assess the effect of the MLT; (3) Results: Changes in the central retinal artery equivalent (CRAE) (*p* < 0.001) and central retinal vein equivalent (CRVE) (*p* = 0.002) parameters were observed. CRAE and CRVE decreased under MLT and increased under the placebo condition from before to after. However, the baseline values of the participants already differed significantly before the application of any therapy, and the variation in the retinal vessel diameters was already large in the baseline measurements. This suggests that the observed effect results may only reflect naturally occurring fluctuations in the microcirculation and not the effect of MLT. Furthermore, no significant effects were observed in any other investigated parameters; (4) Conclusion: Our study with healthy participants finds significant changes in retinal parameters, but the biological variation in the baseline measurements was large to begin with. This suggests that the observed effect results only reflect naturally occurring fluctuations in the microcirculation and not the effect of MLT. However, in the future, larger studies in which MLT is applied for longer periods and/or in patients with different diseases could discover the physiological impacts of this type of therapy.

## 1. Introduction

The disturbance of cardiovascular physiological processes is the foundation of cardiovascular morbidity and mortality. There are many risk factors, including high blood pressure, high blood cholesterol, smoking, diabetes, and others, that contribute to the development of cardiovascular disease (CVD). Aging reduces the elasticity of arteries, which decreases the flow of blood and oxygen, and can result in a permanently high blood pressure [1]. High cholesterol intake increases the level of LDL cholesterol and contributes to atherosclerosis development, resulting in narrowed arteries and hypertension [2]. Moreover, smoking increases cholesterol, LDL, and triglycerides, but decreases HDL [3]. The status of diabetes increases the level of oxygen radicals and reduces the level and bioavailability of NO, which decreases the elasticity and narrowing of blood vessels [4].

Yoga and meditation practices are considered to be non-invasive approaches to improving cardiovascular health [5,6]. These types of Vedic techniques also include light therapy. However, evidence and claims about the effect of light therapy are limited and insufficient [7,8,9].

It is suggested that light treatments produce biological effects in molecules, living cells and tissues [10,11,12,13,14,15].

One of the most common health issues treated by phototherapy is wound healing. Red light is typically used for wound healing due to its increased penetration through tissue and its lower absorption by hemoglobin and melanin [10]. The irradiation of human fibroblasts with red light (λ = 628 nm) leads to an increase in DNA and RNA synthesis with gene expression upregulation, with most of them playing a role in increasing cell proliferation and suppressing apoptosis [11].

Other proteins whose activity can be affected by phototherapy include superoxide dismutase (max in 644 nm), glucose oxidase (464 nm), cholesterol esterase and cholesterol oxidase, and lipase (400 nm) [12,13].

The effectiveness of therapy that uses wavelengths in the visible region has been shown for various medical conditions, such as dengue fever, insomnia, diabetes, psychiatric illnesses, hypertension, seasonal affective disorder (SAD), immunity, hyperacidity, cutaneous wound healing, chronic joint diseases and inflammation [12,14,15,16,17,18,19].

Thrombocytopenia is a typical symptom in patients with dengue fever. Chromotherapy by red-colored radiation (644 nm) has been shown to inhibit platelet degradation [15]. Furthermore, the limited options for treating insomnia during pregnancy has led to the application of chromotherapy. It has been shown that the color turquoise (495 nm) improves sleep, and decreases fatigue and drowsiness during pregnancy [16]. In turn, a light therapy with green, yellow or orange light can be helpful in controlling diabetes [19]. Bright Light Therapy has been proven to be an adequate countermeasure for SAD and other mental health disorders [18]. A detailed discussion of the different methods is summarized in a paper by Azeemi and coworkers [17]. Despite the presentation of different studies focused on light therapy, most of them struggle with problems regarding the sample size and heterogeneity, so further research is needed [8].

Maharishi light therapy uses Vedic technology, which was first applied more than 30 years ago by Joachim Roller, an apprentice of Maharishi Mahesh Yogi [20,21]. Following Maharishi’s guidance, a jewelry designer, Joachim Roller, developed gem beamers. Since 2007, the gem beamer technology has been used around the world. Maharishi light therapy (MLT) focuses on light passing through gems (such as diamonds, emeralds, and rubies) and it is applied to specific areas of the body. People report that the therapy is enjoyable, deeply relaxing, and refreshing for their mind and body [20,21]. To date, however, only a limited number of articles have evaluated the impact of light therapy on physiology, and none have focused on Maharishi light therapy. The lack of knowledge about Maharishi light therapy is evident and, as Travis et al. noted, “an assessment of the impact of light therapy on the physiology of the human body is necessary” [22]. This pilot study investigated the effects of MLT on physiological parameters within a triple-blinded randomized, crossover study. We assessed hemodynamic parameters, heart rate variability (HRV), blood pressure variability (BPV), and microvascular responses in healthy participants.

## 2. Materials and Methods

The investigations of this study were performed at the Medical University of Graz, Austria. The study was submitted to and approved by the Ethics Committee of the Medical University of Graz, Austria (EK: 30-515 ex 17/18). Data collection was performed in accordance with good clinical practices and following the WMA Declaration of Helsinki (2013). Every participant received detailed information about the study protocol and provided written consent.

### 2.1. Participants

In total, 30 (14 males and 16 females) healthy, non-smoking participants were enrolled in this study. They were between 23 and 71 years of age (46 ± 18 years), of 160–185 cm in height (170.9 ± 7.8 cm), and 51–130 kg in weight (71.0 ± 18.7 kg) (Table 1). The exclusion criteria were individuals who smoked, consumed alcohol on a regular basis, had psychological problems, had heart disease, were on medications that influence cardiac parameters (e.g., beta blockers), or were pregnant.

### 2.2. Study Design

This was a triple-blind, randomized, crossover study. Neither the administrator of the light therapy nor the participant receiving the treatment knew about the order of the MLT vs placebo intervention. The measurements took two days (48 h, 24 h per study condition). Participants were randomly assigned to two appointments within two successive days. The participants who received MLT on the first day crossed over to the second intervention (placebo) on the second day and vice versa. All measurements were obtained within two days (48 h). The placebo intervention was nonrecognizable from MLT with real gems. The study was conducted to investigate the effect of MLT on the physiological parameters and microvasculature. Data analysis was performed offline by a person (R.N.) who had no knowledge of the treatments/condition (MLT or placebo) on a given day. Randomization was performed by using a free, demo version of online software https://www.randomizer.at (accessed on 1 April 2019).

### 2.3. Light Therapy Application Device

Gems that emitted different colors of light were used. A device comparable to the one that administered the MLT was also made, but only LED lights of similar colors to the ones used in the MLT were used. It was not possible to differentiate the gems- vs the placebo light-administering device.

#### 2.3.1. Light Therapy Pens

The MLT pens were around 1.5 cm in diameter and 15 cm long, powered by a battery. The light passed through 13 different gems: amber, amethyst, blue sapphire, carnelian, cat’s eye, coral, diamond, emerald, green tourmaline, pearl, ruby, yellow sapphire, and zircon. The light was projected through the gems to the core of the body (abdomen, chest). The incident lights were focused in circles, whose size indicated how far the pens were placed from the participant’s body.

#### 2.3.2. Placebo Light Pens

The placebo light pens were of the same shape, color, and material as the authentic light therapy pens. They projected light through colored glass instead of gems. The light was similar in color and diameter to that in the light therapy pens, and the real application device and placebo version could not be distinguished from each other.

### 2.4. Light Therapy Protocol

As mentioned in the study design, the participants were given two appointments, one for the MLT with real gems and one for the placebo intervention. Each intervention was 24 h apart and each participant was investigated under both conditions, using MLT and placebo light therapy, in a randomized crossover design. The MLT light therapy was performed by an experienced practitioner (E.B.), who had no knowledge of who received which intervention (MLT or placebo) on a given day.

**Figure 1:**
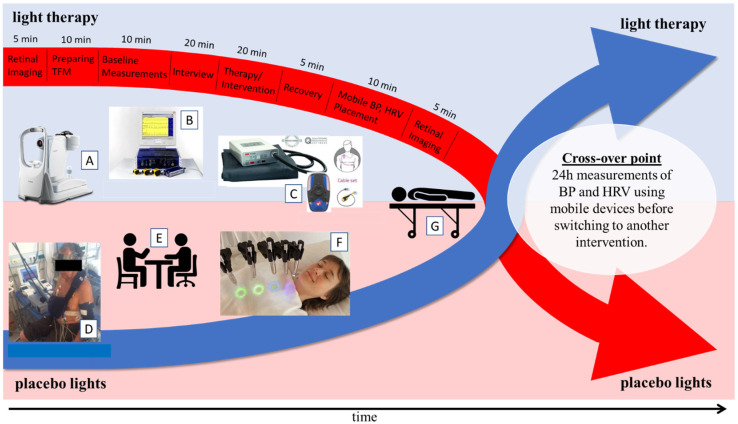
Simplified light therapy study protocol. The red and blue arrows represent the crossover of the two interventions (MLT or placebo light) of the study group. The red arrow is used to display the flow of the protocol procedures and the respective time slots. The individual small images (**A**–**G**) in the core of the picture display the equipment and procedures used during the experiment (explained below). The crossover point describes the timing between the switch of therapies. (**A**)—retinal camera used to capture retinal images, (**B**)—task force monitor used to measure all physiological parameters, (**C**)—mobile devices used to measure 24 h variability in blood pressure (**left**) and heart rate (**right**) (detailed in Section 2.6), (**D**)—baseline measurements of physiological parameters using TFM, (**E**)—interview during the protocol, (**F**)—therapy/intervention (MLT or placebo light), and (**G**)—recovery in the supine position.

After the participant arrived, they received detailed information about the study protocol and provided written consent. The protocol began with the retinal imaging (5 min). Thereafter, electrodes were placed on the participant’s body, and with lying them in a supine position, the 10 min baseline values of the hemodynamic parameters (TFM, Task Force Monitor) were recorded. The protocol continued with an interview with the practitioner of the study (20 min) (information not included in manuscript). Following, the participants were exposed to either MLT or placebo light intervention, using the same apparatus; however, instead of gem beams, LED beams with the same light colors were used (20 min). The administration of the two conditions was randomized and blinded, and both conditions (–MLT/placebo light) were administered on successive days. Physiological measurements using TFM (epochs T1–T25) were collected during the whole intervention, as well as during recovery in the supine position (5 min). After recovery, BP and HRV devices for continual 24 h measurements were placed on the participant (10 min). The devices measured the BP and HR of the participants over the following 24 h until the participant underwent the alternating intervention. These measurements enabled the evaluation of a potentially longer light therapy impact. Before the participant left, the second retinal imaging procedure was performed (5 min). Participants returned the next day (after 24 h) to receive the other condition/intervention. The procedure for the light therapy protocol is described in Figure 1.

### 2.5. Physiological Measurements

All measurements were collected using a Task Force Monitor^®^ (TFM, CNSystems, Graz, Austria). BP (upper arm oscillometry and finger plethysmography), HR (3-lead ECG) and thoracic impedance were measured for the purposes of hemodynamic monitoring. For detail of the electrode’s placement, see Trozic et al., (2020) [23].

The collected physiological data were analyzed by calculating the means of 30 s epochs. The last 30 s of the baseline were taken as a reference (epoch T0), and then analogous with the light intervention, which lasted between 13 and 30 min. In order to remain consistent, the first 25 epochs of the MLT/placebo were taken: T1-T25. Only means with more than 85% valid values (more than 25 s) were taken, and the rest were set to missing values in order to reduce the bias that could have arisen in any of the analyses [24]. The means and the plots of the epochs were generated using MATLAB R2018a (Version 9.4.0, The MathWorks Inc., Portola Valley, CA, USA). The totals of the 26 epochs were then analyzed using SPSS (Version 26.0, SPSS Inc., Armonk, NY, USA).

### 2.6. Heart Rate Variability and Blood Pressure Variability Measurements

A portable ECG-measurement device (eMotion Faros, Biosign, Ottenhofen, Germany) was used to record the participants’ ECG over the course of 24 h after each intervention (MLT or placebo). These data were then applied to a long-term (24 h) HRV analysis. A similar dataset with the participants’ BP measurements (TM-2430, A&D) over the 24 h after each intervention was also acquired and analyzed.

The HRV data were analyzed by removing any large motion artifacts with the help of the Symlets 4 (sym4) wavelet transformation. Then, the Pan-Tompkins algorithm was used to detect the R-peaks in the 24 h ECG signal and build the normal-to-normal intervals (NN intervals) of the participants [25]. The NN intervals were analyzed with statistical time domain methods—e.g., SDNN (standard deviation of all NN intervals), SDSD (standard deviation of differences between adjacent NN intervals), etc. [25]. The parameters that were analyzed are all in the time domain as the frequency domain parameters and are more prone to errors due to artifacts. Furthermore, the time domain parameters correlate directly to frequency domain parameters and therefore can be used as a measure for those [25]. The 24 h BPV data were partly analyzed externally. The University of Minnesota provided the sphygmochron data, which contain the MESOR; this is an adjusted 24 h mean, which was obtained by fitting a cosine model to the original data [26].

### 2.7. Retinal Measurements

The retinal images (resolution of 1536 × 1536) of the right eye were obtained from each of the participants before and after each intervention, as indicated in the protocol. To capture the optic disc-focused retinal images, a non-mydriatic digital retinal camera, the Canon CR-2 (Canon Medical Systems Europe B.V., Zoetermeer, The Netherlands), was used. Retinal images were arranged, organized, and prepared for analysis. A trained operator, without any previous knowledge about the details of the study, used the semi-automated MONA REVA software (VITO, Mol, Belgium; [27]) to analyze the retinal images. The software automatically processed the retinal images and analyzed the diameters of retinal microvessels in areas 0.5 to 1 of the optic disc radius from the optic disc margin. Post-processing, including double thresholding, blob extraction, the removal of small connected regions, and filling holes, was performed. Subsequently, the vessels (arterioles, venules) were checked, corrected, and labeled by the grader. The Parr–Hubbard–Knudtson formula, which uses the 6 largest retinal arterioles and the 6 largest retinal venules, was used to calculate three retinal parameters: central retinal artery equivalent (CRAE), central retinal vein equivalent (CRVE), and artery-to-vein ratio (AVR) [28].

### 2.8. Statistical Analysis

The physiological parameters obtained during the study were first analyzed by the Shapiro–Wilk normality test to check the distribution of the data. Afterward, repeated measures one-way and two-way ANOVA, and analyses of the effects of the different covariates on the linear model, were used. The repeated measures ANOVA was performed for all the participants pooled together and once additionally for sex as a between-subject factor. Three covariates were additionally considered for the analysis: age, weight, and height. Any between-subject factors and covariates that were found not to be statistically significant were removed from the further analysis. All statistical tests were performed with the proper assumptions checks (e.g., Shapiro–Wilk test for normality), and any inconsistencies were removed. The data are presented as means ± standard deviation.

The data from the 24 h HRV and BPV measurements were checked for normal distribution with the Shapiro–Wilk test. The HRV parameters were non-normally distributed and were, therefore, analyzed using the non-parametric Wilcoxon test. The data are presented as means ± standard deviation. Because the BP variability parameters were normally distributed, the standard arithmetic means to perform *t*-tests were used. The data are presented as means ± standard deviation.

The normal distribution of retinal parameters was also analyzed using the Shapiro–Wilk test, and a repeated measures ANOVA was applied to analyze the data; this included *before* and *after,* and the MLT *or* placebo as the repeated measures factor, testing all participants, triple-blinded, under both conditions in a randomized design. To evaluate specific effects, respective post hoc tests were performed, correcting the alpha level according to Bonferroni.

## 3. Results

Thirty participants were recruited for this crossover study. Five participants were excluded from the retinal imaging analysis due to the very low quality of the retinal images obtained from them. Table 1 summarizes the basic characteristics of the study participants.

### 3.1. Physiological Parameters Obtained with the Task Force Monitor

None of covariates age, weight, and height, showed an effect on the linear model. Thus, the parameters were excluded from the analysis. The cardiovascular parameters were assessed at six different time points (T0–T5, see Supplementary Table S1).

Of all the hemodynamic parameters examined, only one significant effect was found for HR (*p* = 0.044), indicating a lower HR (mean ± SD) under the placebo condition than under the MLT condition (mean ± SD); however, the difference in the means was less than 2 bpm (1.616) (Supplementary Table S1)**.**

### 3.2. Heart Rate and Blood Pressure Variability Measurements

No statistically significant results were found in the 24 h HRV and BPV measurements (Supplementary Tables S2 and S3).

### 3.3. Retinal Measurements

In this study, retinal data were available for 25 participants, comparing images taken *before* and *after* the MLT and placebo interventions. Significant differences were found in the change in the CRAE (*p* < 0.001) and CRVE (*p* = 0.002) before and after the MLT/placebo intervention (Table 2 and Supplementary Table S4).

While under MLT the CRAE and CRVE decreased from before to after, the CRAE and CRVE increased under the placebo condition. However, the measures of the participants already differed significantly before the start of any of the interventions. As can be seen in Table 2, the variation in the retinal vessel diameters was large already in the baseline measurements. Hence, the significant effect results from the opposing change in CRAE (and CRVE) under MLT compared to the placebo, which might only reflect naturally occurring fluctuations in the microcirculation, rather than an effect of MLT.

## 4. Discussion

In this study, we investigated the effect of MLT on cardiovascular physiology. We observed that CRAE and CRVE became smaller after MLT, and to the same extent, wider after placebo therapy. However, CRAE after MLT reached values similar to those achieved under the baseline condition before the placebo light therapy. Furthermore, baseline values of the same individual differed to the same extent before the application of any therapy, suggesting that the observed effect is only due to naturally occurring fluctuations in the opposing direction. Furthermore, no significant effects on HRV and BPV were found. To our knowledge, no previous studies have examined the effect of light therapy on retinal microcirculation.

Light therapy, as well as yoga or meditation, is a type of energy therapy in which the belief that there are energy fields that flow through and around your body plays an important role. One recent study investigated the effect of a 4-week cardiac rehabilitation intervention on 2 groups of patients, using typical exercise therapy (control group) and typical exercise therapy plus transcendental meditation (intervention group) to determine both cardiovascular and muscular responses [29]. They observed a significant reduction in systolic blood pressure and a nearly significant reduction in HR; they also observed a significant elevation in the RR interval after 4 weeks of rehabilitation, without interactions between the groups [29]. This study also examined the retinal microcirculation parameters. It included one more follow-up measurement and another rehabilitation group of patients (typical exercise therapy and yoga exercise). However, no significant results were observed during the study, as well as between the different study rehabilitation groups (data not published).

Several previous studies showed a decrease in the HRV, and a switch in the sympathovagal balance to the sympathetic side after light therapy [30,31,32]. We did not observe changes in the HRV parameters.

The narrowing of vessels limits blood flow, hence increasing BP within the walls of the blood vessels [33]. However, we did not find changes in BP in our study. Light therapy in the visible range spectrum can affect photosensitive molecules, such as ATP, superoxide dismutase, or cytochrome C oxidase, which then affect the redox balance in the cells [11,12,13]. Current studies suggest that light therapy leads to an increase in mitochondrial activity, ROS production, as well as NO and, therefore, in vasodilation [11,34,35,36]. We did not find that light therapy had any significant effects on systolic blood pressure (SBP) or diastolic blood pressure (DPB). A pilot study that included 44 hypertensive subjects investigated the effect of laser acupuncture on BP, body weight, and HRV [37]. The low-level laser treatment (90 days, at least 12 treatments per subject) caused a significant reduction in both SBP and DBP [37]. Therefore, in our case, a short period of light therapy and the fact that our study group included only healthy individuals could be reasons for the lack of significant results found. To support the light therapy findings above, Heiss and colleagues, in a randomized crossover study that included 14 healthy male subjects, investigated the 2-day effect of monochromatic blue light or blue light with a filter foil (control light) on cardiovascular health. One light session took 30 min [36]. They saw that monochromatic blue light causes a decrease in SBP and arterial stiffness, and improves endothelial function [36]. Moreover, they also observed increased blood flows and increased levels of nitric oxide species as clear signs of vasodilatation, which is in contrast with the present study. However, our baseline values in the same individual differed to the same extent before the application of any therapy, suggesting that the observed effect is only due to naturally occurring fluctuations. Furthermore, this was not confirmed by any significant effects in other investigated parameters.

Regarding HR, a study that included 7 participants observed a decrease in the HR after 10 min of blue light (456 nm) exposure [30]. Another study investigating free-living trends in sleep and recovery found that the mean HR was significantly lower the night after light therapy [38]. The study included 12 athletes, who were exposed to visible red (660 nm) and near-infrared (NIR, 850 nm) light in an average of 8.5 ± 7.5 sessions/athlete, while one session took 20 min. No more than 2 sessions and no sessions on consecutive days were allowed. Distinct from their study, we observed a higher HR after MLT (*p* = 0.044). However, the significant effect was so small that the difference in the means was less than 2 bpm (1.616), so it could be easily caused by any small variation during the flow of the protocol. Unfortunately, the statistical tests only detect relative differences and no absolute values, such as 2 or 20 bpm. Therefore, these results must be interpreted very carefully and should be repeated in future research.

No significant effects on HRV and BPV were found in our study. The crossover study of Travis et al. found that MLT had a significant effect on the subjective feeling of wellness in 18 individuals with experience in meditation [22]. The design of their study was similar to ours, but all subjects were long-term meditation practitioners, and the favorable results of the study could be easily caused by the effect of meditation rather than by MLT itself.

The same study that observed a decrease in HR after 10 min of exposure to blue light (456 nm) also noted a decrease in HRV [30]. Yuda et al. found a significant decrease in high frequency (HF) and an increase in the low frequency (LF)/HF ratio as an indicator of reduced parasympathetic activity; this was due to the effect of 6 min of blue, red, and green light exposure [31]. Another study included 20 participants and found that 10 min of exposure to red light increases LF/HF ratio and LF, hence increasing sympathetic activity [32]. On the other hand, blue light causes a reduction in LF and the LF/HF ratio, and an increase in HF causes more cardiac relaxation via parasympathetic activity [32].

While we could not find any additional studies that examined the effects of light therapy on HRV and BPV, the available literature from yoga studies show that yoga exercise leads to a significant increase in HRV parameters, especially those associated with the vagal tone [39,40,41]. Khattab and colleagues observed an increase in HRV as the effect of a 5-week (once week/90 min) yoga program in 11 healthy (7 women and 4 men, mean age: 43 ± 11; range: 26–58 years) study participants [39]. Similarly, Papp and colleagues noticed increased vagal tone and reduced sympathetic activity after an 8-week yoga program [40]. In agreement with the previous two studies, a shift in the sympathovagal balance in favor of parasympathetic dominance was observed after meditation practice (once per day, 4 times per week for one year) [41]. However, most studies investigating the effect of yoga on HRV are completed in India, struggle with sample size, are poor in design and quality, and use a range of heterogeneous measures [41,42,43,44]. Overall, the effect of yoga on HRV parameters appears to be beneficial, and shifts the autonomic regulation on the parasympathetic side; however, more research, especially from western countries, is needed in order to confirm these findings.

## 5. Limitations

There are some limitations to this study: (1) We do not have detailed information about the gem lights used for the MLT or knowledge about wavelengths. It is difficult to compare our results with previous research and an overall interpretation of the results must be performed carefully; (2) According to previous studies about the effects of light therapy on human health, the majority of the studies applied light for longer periods of time than our study (the length of the intervention in our protocol was set according to the recommendations of the MLT practitioner), and it is possible that we did not see changes in any other parameters because the application time of the light was short; (3) While our study investigated cardiovascular physiology parameters in healthy participants, most studies used light therapy as a treatment (e.g., depression, hypertension, wounds healing, etc.), when the effect of light therapy could be possibly more pronounced by the imbalance of the body; (4) As it is presented in the results section, the significant effects might only reflect naturally occurring fluctuations in the microcirculation, rather than an effect of MLT, and, therefore, these results need to be interpreted very carefully; and (5) The observed caliber of the retinal microvasculature could also be influenced by errors introduced while measurements were being taken. We do not believe that this is the case, as all the retinal measurements were carried out by the same person (A.S.).

## 6. Conclusion and Future Directions

The present pilot study observed changes in CRAE (*p* < 0.001) and CRVE (*p* = 0.002). CRAE and CRVE decreased under MLT and increased under the placebo condition from before to after. Hence, the significant effect results from the opposing change in CRAE (and CRVE) under MLT, compared to the placebo. However, because the baseline values of the participants already differed significantly before the application of any therapy, and the variation in retinal vessel diameters was large already in the baseline measurements, the observed effect results may only reflect naturally occurring fluctuations in the microcirculation and not the effect of MLT. Thereby, the results observed in the present pilot study should be treated with caution. No other significant changes in the measured physiological parameters were found to support the findings related to retinal microcirculation. It is possible that the application of the MLT was of a rather short duration.

The present pilot study investigated the effects of MLT on healthy volunteers. In the future, larger studies, in which MLT is applied for longer periods of time and/or in patients with different diseases (e.g., depression, hypertension), could discover the physiological impacts of this type of therapy.

## Figures and Tables

**Table 1:** Characteristics of the study participants (*n* = 30). Data give mean ± SD (range).

Characteristics	Males = 14		Females = 16	
Age (years)	(46 ± 20)	23–71	(46 ± 17)	24–71
Height (cm)	(176.7 ± 6.2)	166–185	(165.8 ± 5.0)	160–179
Weight (kg)	(82.5 ± 19.5)	56–130	(61.1 ± 10.9)	51–85
BMI (kg/m^2^)	(26.5 ± 6.8)	20–45	(22.2 ± 3.6)	19–32

**Table 2:** The results of retinal microcirculation measurement. The means, and standard deviations of the retinal parameters of participants (*n* = 25). CRAE (central retinal artery equivalent), CRVE (central retinal vein equivalent), and AVR (artery-to-vein ratio). Data are shown as means (SD).

Retinal Parameter	Intervention	Before	After	F-Value, *p*-Value
CRAE (µm)	MLT	148.9 (18.1)	145.6 (16.3)	F(1,24) = 17.004, *p* < 0.001
	Placebo	145.5 (18.1)	147.5 (17.5)	
CRVE (µm)	MLT	222.7 (20.6)	218.3 (19.8)	F(1,24) = 11.999, *p* = 0.002
	Placebo	219.5 (22.1)	221.9 (20.2)	
AVR	MLT	0.668 (0.047)	0.668 (0.051)	F(1,24) = 0.069, *p* = 0.796
	Placebo	0.663 (0.049)	0.665 (0.053)	

## Data Availability

All data are contained within the article.

## Supplementary Materials

The following supporting information can be downloaded at: https://www.mdpi.com/article/10.3390/jcm12062229/s1, Table S1: Overview of the cardiovascular parameters. Displayed as mean (±SD) of the first 6 epochs—the last 30 s of baseline (T0) and the first 2.5 min of the light therapy (T1–T5), of all participants, pooled together (*n* = 30). HR (heart rate), SBP (systolic blood pressure), DBP (diastolic blood pressure), MAP (mean arterial pressure), SV (stroke volume), CO (cardiac output), TPR (total peripheral resistance); Table S2. The 24 h HRV parameters of two interventions (MLT or placebo light) of the study group. Displayed as means (±SD), z-score and *p*-value. NN50 count (number of pairs of adjacent NN intervals differing by more than 50 ms in the entire recording), pNN50 (NN50 count divided by the total number of all NN intervals), SDNN (standard deviation of all NN intervals), RMSSD (the square root of the mean of the sum of the squares of differences between adjacent NN intervals), SDSD (standard deviation of differences between adjacent NN intervals), SDANN (standard deviation of the averages of NN intervals in all 5 min segments of the entire recording), SDNN index: (mean of the standard deviations of all NN intervals for all 5 min segments of the entire recording); Table S3. An overview of the 24 h blood pressure parameters (mean ± SD) of all participants, as well as the results of the paired *t*-test. It shows the parameters as means ± standard deviation and the results from the paired *t*-tests, presented as the T-score, the degrees of freedom, and the p-value. HR (heart rate), SPB (systolic blood pressure), DPB (diastolic blood pressure), MESOR (midline estimating statistic of rhythm); Table S4. Within-subject effects for the total cohort of participants without between-subject effects or covariates. CRAE (central retinal artery equivalent), CRVE (central retinal vein equivalent), AVR (artery-to-vein ratio).
