# Supplementary material for: Vascular Responses following Light Therapy: A Pilot Study with Healthy Volunteers
Source: J Clin Med. 2023 Mar 13;12(6):2229. doi: 10.3390/jcm12062229 (PMC10054429; doi:10.3390/jcm12062229)
Supplement: Supplementary file 1 [file jcm-12-02229-s001.zip › jcm-2171482-supplementary.pdf]

Supplementary Materials

Table S1. Overview of the cardiovascular parameters. Displayed as mean (± SD) of the first 6 epochs—the last 30 s of baseline (T0) and the first 2.5 min of the light therapy (T1–T5), of all participants, pooled together ($n = 30$). HR (heart rate), SBP (systolic blood pressure), DBP (diastolic blood pressure), MAP (mean arterial pressure), SV (stroke volume), CO (cardiac output), TPR (total peripheral resistance).

Cardiovascular Parameters	Intervention	First 6 Epochs—The Last 30 s (Mean ± SD)						*p*-Value
		T0	T1	T2	T3	T4	T5	
HR	MLT	68 (11)	63 (9)	64 (9)	63 (9)	63 (9)	64 (10)	0.044
(bmp)	Placebo	66 (10)	63 (11)	63 (12)	62 (10)	63 (10)	63 (11)	
SBP	MLT	118 (15)	113 (21)	114 (20)	116 (19)	116 (19)	116 (19)	0.32
(mmHG)	Placebo	120 (19)	116 (23)	112 (17)	112 (16)	112 (16)	113 (16)	
DBP	MLT	73 (14)	68 (17)	69 (18)	70 (17)	70 (17)	69 (17)	0.671
(mmHG)	Placebo	75 (14)	71 (15)	69 (15)	69 (14)	69 (14)	70 (14)	
MAP	MLT	91 (14)	86 (18)	87 (18)	89 (16)	89 (16)	88 (16)	0.743
(mmHG)	Placebo	92 (15)	89 (18)	86 (15)	86 (15)	87 (14)	87 (14)	
SV	MLT	89 (26)	92 (28)	90 (28)	90 (28)	90 (27)	90 (26)	0.329
(ml)	Placebo	90 (28)	92 (29)	92 (31)	93 (32)	91 (30)	92 (30)	
CO	MLT	6 (2)	6 (2)	6 (2)	6 (2)	6 (2)	6 (2)	0.88
(l/min)	Placebo	6 (2)	6 (2)	6 (2)	6 (2)	6 (2)	6 (2)	
TPR	MLT	1293 (454)	1304 (569)	1318 (585)	1378 (610)	1353 (607)	1328 (526)	0.388
(dyne x s/cm^5)	Placebo	1347 (532)	1384 (647)	1334 (574)	1333 (576)	1357 (605)	1342 (594)	

Table S2. The 24 h HRV parameters of two interventions (MLT or placebo light) of the study group. Displayed as means (± SD), z-score and *p*-value. NN50 count (number of pairs of adjacent NN intervals differing by more than 50 ms in the entire recording), pNN50 (NN50 count divided by the total number of all NN intervals), SDNN (standard deviation of all NN intervals), RMSSD (the square root of the mean of the sum of the squares of differences between adjacent NN intervals), SDSD (standard deviation of differences between adjacent NN intervals), SDANN (standard deviation of the averages of NN intervals in all 5 min segments of the entire recording), SDNN index: (mean of the standard deviations of all NN intervals for all 5 min segments of the entire recording).

Parameters	Intervention	Mean (SD)	Z	*p*-Value
NN50 count (total number)	MLT	8208 (6718)	−0.751	0.452
	Placebo	7438 (7311)		
pNN50 (%)	MLT	14.87 (12.60)	−0.956	0.339
	Placebo	12.56 (11.33)		
SDNN (ms)	MLT	220.37 (71.66)	−0.934	0.350
	Placebo	206.5 (68.60)		
RMSSD (ms)	MLT	186.5 (122.14)	−0.114	0.909
	Placebo	166.4 (104.94)		
SDSD (ms)	MLT	186.5 (122.14)	−0.114	0.909
	Placebo	166.4 (104.94)		
SDANN (ms)	MLT	139.91 (50.26)	−0.296	0.767
	Placebo	140.73 (50.13)		
SDNN index (ms)	MLT	143.38 (83.10)	−0.296	0.767
	Placebo	127.14 (64.64)		

Table S3. An overview of the 24 h blood pressure parameters (mean ± SD) of all participants, as well as the results of the paired t-test. It shows the parameters as means ± standard deviation and the results from the paired t-tests, presented as the T-score, the degrees of freedom, and the *p*-value. HR (heart rate), SPB (systolic blood pressure), DPB (diastolic blood pressure), MESOR (midline estimating statistic of rhythm).

24 h Blood Pressure Parameters	Intervention	Mean (SD)	T	df	*p*-Value
MESOR SBP (mmHg)	MLT	131 (14)	0.797	26	0.433
	Placebo	130 (11)			
Mean SBP (mmHg)	MLT	133 (15)	0.911	26	0.371
	Placebo	132 (10)			
MESOR DBP (mmHg)	MLT	77 (7)	−0.09	26	0.931
	Placebo	78 (6)			
Mean DBP (mmHg)	MLT	79 (7)	0.351	26	0.728
	Placebo	79 (6)			
MESOR HR (bpm)	MLT	70 (7)	−0.49	26	0.628
	Placebo	71 (8)			
Mean HR (bpm)	MLT	72 (7)	0.101	26	0.921
	Placebo	72 (8)			

Table S4. Within-subject effects for the total cohort of participants without between-subject effects or covariates. CRAE (central retinal artery equivalent), CRVE (central retinal vein equivalent), AVR (artery-to-vein ratio).

Parameter	Effect		
	**MLT-Placebo**	**Before-After**	**MLT-Placebo x Before-After**
CRAE	$F(1,24) = 0.710$, $p = 0.408$, $\varepsilon = 1$, partial $\eta^2 = 0.029$	$F(1,24) = 0.550$, $p = 0.465$, $\varepsilon = 1$, p. $\eta^2 = 0.022$	$F(1,24) = 17.004$, $p = 0.000$, $\varepsilon = 1$, p. $\eta^2 = 0.415$
CRVE	$F(1,24) = 0.020$, $p = 0.889$, $\varepsilon = 1$, partial $\eta^2 = 0.001$	$F(1,24) = 0.913$, $p = 0.349$, $\varepsilon = 1$, p. $\eta^2 = 0.037$	$F(1,24) = 11.999$, $p = 0.002$, $\varepsilon = 1$, p. $\eta^2 = 0.333$
AVR	$F(1,24) = 0.947$, $p = 0.340$, $\varepsilon = 1$, partial $\eta^2 = 0.038$	$F(1,24) = 0.017$, $p = 0.896$, $\varepsilon = 1$, p. $\eta^2 = 0.001$	$F(1,24) = 0.069$, $p = 0.796$, $\varepsilon = 1$, p. $\eta^2 = 0.003$
